# A Qualitative Study of Nursing Management in Iran

**DOI:** 10.1155/2021/1315734

**Published:** 2021-03-11

**Authors:** Ahmad Kalateh Sadati, Seyed Taghi Heydari, Najme Ebrahimzade, Kamran Bagheri Lankarani

**Affiliations:** ^1^Department of Sociology, Yazd University, Yazd, Iran; ^2^Health Policy Research Center, Institute of Health, Shiraz University of Medical Sciences, Shiraz, Iran

## Abstract

**Background:**

Nursing managers have a critical role at the hospitals. The current study aims to investigate different experiences of nursing managers.

**Method:**

This is a qualitative study that investigates the experiences of 11 nursing managers in Shiraz, Iran. Semistructured interviews and thematic analysis were, respectively, applied for data collection and analysis.

**Results:**

It could be found from the current investigation that nursing managers have a critical role at the hospitals, and their creativities have more impacts on procedures compared to organizational orders. There are four major challenges faced by nursing managers including nursing shortage, structural deficiencies, lack of authorities, and burnout. Although shortage is considered as the most important challenge, there are more emphasizes on the improvement of their controlling power in order to prevent their fatigue and burnout.

**Conclusion:**

This study showed that creativity of nursing managers is the most important factor of system management; also, shortage and deficiencies are recognized as the most significant challenges faced by them. According to the current study, the shortage of nursing staff is the central issue that has to be considered.

## 1. Introduction

Nursing managers play a critical crucial role at the hospitals; therefore, they are considered as the captain of hospitals, and the effective actions of first-line nurse manager lead to the organizational success [1]. A professional nurse manager has perfect actions and has to manage the changes, cultural integrations, retentions, and direction of staff attitudes towards changes of healthcare structures [2, 3]. They also play an important role in establishing creativities in the work place and procedures, job satisfaction, and nurse' retention [4–7]. Significant factors including the improvement of work place empowerment, organizational commitment, and job satisfaction of nurse managers are highly influenced by the management styles of organizations; moreover, they fill the gap between two fields of management and medicine [8]. Although nursing managers have a vital role in improving the work environment, their efficacy is highly affected by their work place [4].

Several researchers have investigated the roles of nursing managers due to its importance. A qualitative research carried out in an Australian critical care unit showed that junior novice would create a submissive identity despite the existence of interprofessional relationships with the lack of individual valuing and low self-esteem [9]. According to the investigations carried out on rural hospitals in Uganda, nurse managers who work in healthcare settings with poor resources are faced with various challenges of quality improvement including limited patient participation, lack of materials, and limited human resources [10]. The transition of nurses into nurse managers is associated with various experiences including struggling in management transition, seeking opportunities for transformation, lack of power to perform commitments, lack of preparation, and leading others by serving [11]. Aitamaa et al. investigated conflicts of practical situations, lack of appreciation, ignoring the problems, and experienced inadequacy [12]. Also, it was shown in an integrative review that there are several challenges associated with transition programs such as lack of transparency of objectives, course content, time frame, and type of provided supports [2]. However, more investigations have to be carried out in different contexts.

During the past decades, many great transformations occurred in the field of nursing in Iran including the establishment of widespread nursing schools at public and private universities, as well as the development of public and private hospitals. These transformations are mainly due to the increasing need for admission beds and made this career become a thriving job in the Iranian society and promoted its social status. Therefore, several high school students choose this major; however, there are many problems associated with nursing in Iran including fatigue and burnout [5, 6], early retirement [13], nursing shortages, job dissatisfaction, poor social positions of nurses, the gap between theory and practice, lack of community-based nursing care, lack of appropriate student recruiting systems, shortages in nursing educational curriculums [14], *lack of nursing positions in policy making* at macrolevels, unemployment, shortage, unclear role, poor image of nursing, and low self-esteem [15, 16]. Although several investigations have been carried out regarding nursing challenges in Iran, there is lack of information about how nursing managers are faced with challenges in these situations. Specifically, there is lack of qualitative knowledge about experiences of nursing managers. Therefore, this study investigates these experiences based on challenges and mainly aims to answer this question: “what are the major challenges of nursing managers?”

## 2. Method

In 2015, a thematic analysis method was carried out in Shiraz, Iran, that investigated head nurses, supervisors, and matrons who worked at public hospitals. All of the cases had at least 2 years of work experience; also, the purposive sampling method was applied in order to study a wide range of related perspectives.

After the objectives of the research were announced to participants, the location of semistructured interview became determined. Two interviews were carried out in the park, two of them were performed at the interviewees' homes, and others were performed at the hospitals' department of nursing managers. The semistructured interviews were conducted based on three main questions:

- Please tell me about your experiences in nursing management.
- What are your challenges in this situation?
- Based on your opinion, what are the consequences of working in this situation?

With verbal consent, interviews were recorded and then became transcribed. Based on saturation criteria, our interviews were saturated with 11 participants (Table 1). It means that, according to the collected and analyzed data, further data collection or analysis has to be carried out. Ideas of other nursing managers except participants and an expert MSS nurse were applied in order to validate the questions of interviews.

Data analysis was carried out using a thematic analysis, which emphasizes on identifying, analyzing, and interpreting the patterns of meaning (“themes”) [17]. Experiences, perspectives, behaviors, and specific practices of participants were investigated using this method [1]. Six phases of Braun and Clarke method were applied in this study [17]. First of all, the explicit and implicit contexts of data were explored [18]. Generating the initial codes, combining codes into overarching themes, looking at how the themes support the data and the overarching theoretical perspective, defining each themes, investigating the captured data aspects, interesting things about the themes, and performing the process of “member checking” [17] were carried out in the next step.

Trustworthiness is considered as the main factor in qualitative researches. The acceptability and usefulness for stakeholders made this study become closer to this criterion [19]. Observations of participants and member checking were carried out in order to ensure the credibility of current study, while transferability was ensured through connecting the cultural and social contexts of data collection. Also, results of the research study and processes of data collection and analysis were examined and confirmed by a nonparticipating researcher to ensure dependability. Finally, it should be noted that audit trial and reflexivity were applied for conformability.

## 3. Research Ethics

A voluntary participant contribution was carried out for current research; also, data were remained confidentiality during the research, and the codes of Declaration of Helsinki and American Sociological Association were observed [20, 21].

## 4. Results

Nursing shortage is considered as the main challenge in current investigation. This is due to the fact that everybody tries to perform the obligations of nurses including physicians, patients, and their relatives, and it leads to the weak action of nurses, patients' dissatisfaction, and cumulative pressures. Therefore, these factors would have a significant impact on the nurse manager in a nondynamic system with increased power and advances in the field of nursing management. Although participants believed that nursing management plays a vital role at the hospital, they did not have a clear view about their management and faced with several unpredictable challenges. There are several demands at the hospitals without any specific resources; therefore, they should perform the management procedures based on their creativity rather than organizational orders. Many of the management issues that had to be carried out by participants were not included in their duties, and they had to solve some problems during the afternoon and night shifts based on their creativities. These challenges include failures, lack of personnel in other sectors, and responding to complaints. Nursing managers should stay calm, control the system without any stress, and apply a holistic approach. However, they did not have enough authority to manage everything, and this led to the chronic boring of our participants.  Table 2 shows four themes related to the participants' experiences including nursing shortage, structural deficiencies, lack of authority, and burnout.

### 4.1. Nursing Shortage

The main concern of all participants was nursing staff shortage.

*“The biggest and biggest problem in nursing is the problem of shortage of nursing staff. That is, our human resources are not good and the layout of our human resources is not good either. Sometimes it's possible to have a good amount of power in one part, but if we want to consider the experienced staff or the quality of the work, we cannot have it in our arrangement”* (participant no. 8).

In this condition, nursing managers would face this problem during all shifts. Replacement of deficiencies is considered as the main challenge, which is faced by managers at the beginning of the shifts and is resulted from the fact that nurses of unrelated sections without required expertise would be sent to the needed sections and it leads to dissatisfaction. More importantly, a shortage of personnel makes it more difficult for managers to define the number of shifts per week for nurses.

*“Due to the shortage, we have not been able to provide nurses satisfaction. Thus, the nursing system causes dissatisfaction. Why? Because they become psychologically unsatisfied in addition to physical works”* (participant no. 1).

According to the abovementioned reasons, the shortage of nurses would have an adverse effect on the work quality as a result of a lack of power. The new inexpert nurses would think that the quality of work does not have a significant importance, and this would result into the shortage of expert nurses in the long-term and, consequently, would reduce the quality of nursing care.

*“The strength forces are also low, and we really have a shortage of nurses, which increases the number of personnel and shifts, and pushes the staff. This also impacts on the knowledge and effectiveness of nurses and consequently, leads to faults and missing”* (participant no. 7).

### 4.2. Structural Deficiencies

Structural deficiencies mean that there are some chronic problems that have not been solved yet. Due to the fact that solving these problems has to be carried out by nursing managers, specifically the night shift, they would have too much mental stress. These deficiencies include the shortage of bed in some wards, equipment failure or shortage, canceling some surgical operations, and weak support for upstream managers.

*“During the night shifts, the clinical supervisor is responsible for most of the problems, and everything has to be referred to them, while the hospital manager and matron do not have these responsibilities. There are more management issues, especially managing other non-nursing parts, during the night shifts. These issues include the absence of physician, not doing sonography by sonographers, shortage of trolleys, equipment or installation failures, etc. The supervisor is responsive and has to solve all of these problems”* (participant no. 3).

Weak educational system was recognized as the most important structural deficiency. Participants believed that nursing educational system does not have a high quality. There is a gap between current needs and old educational systems, and quantity is more emphasized in some cases; therefore, there are several graduated nurses who participate in training courses after being hired at the hospitals.

*“Weak and inappropriate training, which is much lower than the world's standards, is recognized as another challenge in this field For example, nursing students who participate in the hospital's practical education do not have an active presence. They spend much of their time at room and present conferences. They do not have an active practice with patients”* (participant no. 5).

One of the participants believed that nursing care does not include technical expertise, but it should have a different worldview about care and treatment processes. Making this worldview is only carried out through education.

*“If anyone asks me that “What is the first thing in nursing?” I would repeatedly imply the education, training, education, and training. This is not included in the teaching of electrocardiography, ventilator, etc. We need to change our worldview and visions. If we change them, we can go after those trainings. But these are not taught and there are lack of education, of awareness, of culture, etc.”* (participant no. 4).

### 4.3. Lack of Authority

Participants believed that they did not have enough authority. It is supposed that nursing managers, specifically matrons and supervisors, have a high organizational authority, while this is not true practically and they are not able to execute the commands correctly. Many of the processes of decision-making are carried out based on nursing administrators; however, this is against nursing managers decisions. For example, nursing administrators or other higher managers in the health system decide about the transfer of nurses to other hospitals without paying attention to nursing managers' decisions. Therefore, it could be said that some main decisions are made by external powers and it makes nursing mangers feel they are powerless.

*“But it does not undermine the integrity of the system as well as the power of management. For example, a staff member brought me a letter from director of the university and put it on my desk and told me: “release me as soon as possible I want to go.” I disagreed and she brought her husband and he told me that you do not need to sign because I've gotten the president's sign. They all discouraged us all because we did not actually have any executive power. I encountered a headache about her fault, and she told to the higher manager of nursing in the province that matron stresses me, and she accepted it. This means that I did not have enough power”* (participant no. 1).

These statements imply the lack of authorities and the existence of other powers that significantly impact on their performances. Sometimes, this powerlessness would be felt more than other times, for example, participant no. 5 was a head nurse and stated as follows:

*“As one of the middle managers of our organization, I think that I do not have the power to have an effective management. Here, a nursing manager does not have any executive power. We do not have the option to secure the budget and even buy a small device such as an optional table head and, more importantly. Also, we cannot choose the forces we need to work in our unit in accordance with the conditions, expectations, and nature of the patients we deal with”* (participant no. 5).

Therefore, nursing managers may not be able to control different situations perfectly and become confused when they face with faults of their staff. All of the abovementioned deficiencies lead to the disability of nursing managers in controlling the system using appropriate approaches. For example, if managers see personnel's faults and decide to punish them, they may face with different threats and complex conditions; therefore, this leads to their weak management. On the other hand, they cannot leave their responsibilities and attempt to report the faults of staff that influence the patients' lives.

### 4.4. Burnout

Nursing managing is a boring task. Managers have to solve problems many of which are not their tasks. They are responsive for everything and may sometimes face with serious challenges. Participants believed that their management was associated with pressures that reduced their potential. Managers apply continuous management approaches, and it leads to their fatigue and, consequently, burnout.

*“One of the main parts of this exhaustion is psychologically and occurs in different types. Generally speaking, this is a difficult job associated with too much pressure. For example, a nurse is only dealing with a patient, while the head nurse must pay attention to both the patient's issues and the problems of his subordinate forces. The supervisor indirectly addresses the patient's problems and controls more forces. Therefore, these pressures would be increased significantly because they are multi-faceted and the organization is not able to meet the needs of patients. Nursing management is a hard, complex, and exhausting task”* (participant no. 3).

In this condition, managers feel a lot of psychological and physical pressures. They get burned out and become less interested very soon, and, consequently, they may even act impolitely. Of course, this is mainly due to the shortage and occurs at all levels. Although managers have a high motivation, they continuously become less commonplace, and this makes them think of leaving the work place.

*“I'm thinking about the hardships and benefits of this job and the people I really love and enjoy working with them”* (participant no. 5).

## 5. Discussion

**Figure 1:**
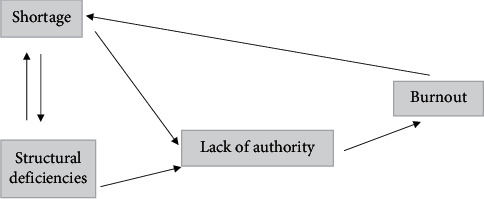
The shortage and structural deficiencies are the main factors that lead to the lack of authority and burnout among managers and, consequently, make them leave the work place.

Results of current research showed that the experience of participated nursing managers was based on two important factors that were beyond their control: shortages and structural deficiencies. Also, authority and burnout had considerable impacts on their performances. However, we believe that shortage should be considered as the most important factor (Figure 1).

Our findings are in accordance with other studies because nursing shortage is also considered as the main factor in other investigations [14, 20, 21], which leads to several problems such as fatigue, dissatisfaction [22], and burnout [23, 24]. Also, it increases the pressures for nurses and determines the quality of nursing care processes. Inappropriate transfer of nurses resulted from the weak work environments and would lead to the entry of new inexpert nurses without enough knowledge and expertise to specific sections and, consequently, dissatisfaction of themselves, patients, and managers [25].

Structural deficiency is considered as another problem, which is influenced by to the general features of developing countries. There are several studies that investigate this challenge in health systems. Sadati et al. showed that the distorted relationships between doctors and patients resulted from inefficient structures [26]. Furthermore, it seems that nursing does not play a vital role in the process of administrative decision making in Iran's health system [16]. Aitamaa et al. [12] carried out another investigation in which the experience inadequacy was studied and found that it could lead to the managers' lack of authority; also, it was showed that this problem could be solved through providing proper nursing conditions. Moreover, organizational support was emphasized in another study that investigated nurse managers who worked with HIV- and/or TB-infected parents [27]. Patrick and Laschinger showed that a combination of empowerment and perceptions of organizational support was a significant predictor of middle-level nurse managers' role satisfaction [28]. We found that the main problem is that the new nursing staff did not have enough knowledge and skills to start their career. Also, it was shown that there was a lack of transparency among nursing students [2], which has led to a gap between these students and nurses who work at the hospitals.

Results of the investigation carried out by Kahn [29] and Job Demands–Resources model [30] revealed that the improvement of nursing managers work engagement requires more organizational supports. According to Job Demands–Resources model, the imbalanced demand for labor and resources causes pressure for managers [30]. Three themes in this study showed that the major reason of work stress is imbalanced demands and resources. It was also found that nursing managers should have more power and authority. It could be said that based on the abovementioned theory and findings that power is not in a balanced state in the healthcare systems, this is considered as an important challenge.

Generally, nursing managers investigated in current study need more authority in order to perform their responsibilities more perfectly. Nurse turnover is considered as a serious issue in Iran [31, 32], which has to be regarded by policy makers and nursing leaders. It is believed that our participants perform management procedures using the constructive/positive conflict management approaches model, which was developed by Labrague et al. instead of destructive/negative conflict management approaches [33]. In fact, they managed the system through a creative approach. Nursing managers work in a sociocultural work place in Iran. As Sadati et al. showed the processes of nursing works during COVID-19 in Iran, this type of work place is associated with metaphysical components that help people to work in difficult situations [34].

According to our findings, organizational managers have to pay more attention to the nursing managers. In current study, providing required resources is in priority. Health policy makers have to understand the importance of using nursing managers and consider it in policy making procedures. Therefore, providing more authority and organizational supports would be very beneficial.

## 6. Conclusion

This study showed that nursing managers would experience many shortages, lack of authority, structural deficiencies, and burnout. In fact, burnout resulted from imbalanced factors of other parts, while lack of authorities would make managers feel that they actually can not do anything. Therefore, policy makers have to pay more attention to the needs of nursing managers and consider them in the policy making procedures. According to current study, considering the shortages and promoting the knowledge and skills of new registered nurses are very important.

## 7. Limitation

Data collection was very difficult because all of the participants had to do their nursing managing duties. These findings could not be generalized because of their qualitative natures.

## Figures and Tables

**Table 1:** Characteristics of participants.

Id	Age	Gender	Literature	Position
1	35	F	B.Sc	Matron
2	34	F	B.Sc	Assistant of matron
3	37	F	B.Sc	Supervisor
4	53	F	B.Sc	Supervisor
5	35	F	M.Sc	Head nurse
6	35	M	M.Sc	Head nurse
7	36	M	Ph.D. candidate	Supervisor
8	51	M	B.Sc	Supervisor
9	41	F	B.Sc	Supervisor
10	42	F	B.Sc	Supervisor
11	45	F	B.Sc	Assistant of matron

**Table 2:** Initial codes, subcategories, categories, and themes extracted from data.

Initial codes	Subcategories	Categories	Theme
One of the major problems is the shortage of nursing staff.	Shortage of staff	Shortage	*Nursing shortage*
I myself was a staff and I know that the importance of force is the most important			
Another problem with our work is the lack of experts.	Shortage of experts		
We are implementing clinical governance and accreditation standards, why? While we do not have nursing for care.	Multidimensional roles	Role	

We have not only a shortage of staff but also a shortage of equipment	Equipment shortages	Infrastructure shortage	*Structural deficiencies*
We sometimes face with shortage of beds	Bed shortage		
In the night and night shifts, which are not anyone, neither hospital manager nor matron, everything is referring to clinical supervisor	Management problems		
Nurses today need to provide more services, educate the patient, and much more functions	More expectations of nurses	Educational deficiencies	
The education system does not pay much attention to new needs of nursing students.	Educational deficiencies		
If anyone asks me what the first thing in nursing is, I'll repeatedly and repeatedly say education, training, education and training. This is not included to teaching of electrocardiography, ventilator, and so on.	Need to trained nurses		

I do not have enough power.	Feeling of no authority	Nothing authority	*Have no authority*
I think that I do not have the power to act effectively to manage affairs.			
They (other higher managers) release the staff without consulting with us.	Nothing authority in practice		
Sometimes staffs don't obey us			
We do not have organizational authority.	Nothing have organizational authority	Organizational authority	

A major part of this exhaustion is psychological, because of a hard job is, in every trace you have, there is a lot of pressure on one person.	Psychological pressure	Psychological pressure	*Burnout*
I have to bear this responsibility for at least 3 years, 4 years, and I think I'm tired and I can't, then.	Fatigue	Fatigue	
Sometimes repairing medical equipment will take a long time and nursing manager will get tired of both the main and more important tasks.			

## Data Availability

The datasets used and analyzed during the current study are available from the corresponding author on reasonable request.
